# Observing the Ocean Submesoscale with Enhanced-Color GOES-ABI Visible Band Data

**DOI:** 10.3390/s19183900

**Published:** 2019-09-10

**Authors:** Jason K. Jolliff, M. David Lewis, Sherwin Ladner, Richard L. Crout

**Affiliations:** U.S. Naval Research Laboratory, Stennis Space Center, Hancock County, MS 39529, USA

**Keywords:** ocean remote sensing, ocean color, coastal ocean processes, colorimetry

## Abstract

Ocean color remote sensing has long been utilized as a fundamental research tool in the oceanographic investigations of coupled biological-physical processes. Despite numerous technical advances in the application of space borne ocean-viewing radiometers, host satellite platforms in a polar-orbiting configuration often render the temporal frequency of sensor data acquisition insufficient for studies of ocean processes that occur within increasingly smaller space-time scales. Whereas geostationary ocean color missions are presently the exception (GOCI) rather than the rule, this paper presents a method to convolve ocean reflectance data obtained from contemporary ocean-viewing multispectral radiometers (VIIRS, OLCI) with spectrally-limited Advanced Baseline Imager (ABI) data obtained from the GOES-R meteorological satellites. The method, Chromatic Domain Mapping (CDM), employs a colorimetry approach to visible range ocean reflectance data. The true color space is used as a frame-of-reference that is mapped by the dedicated yet temporally sparse ocean color sensors; coincident and spectrally coarse information from ABI is then used to estimate the evolution of the true color scene. The procedure results in very high resolution (~5 min) true color image sequences. Herein, example CDM applications of rapid frontal boundary evolution and feature displacement in the Gulf of Mexico are presented and future applications of this technique are discussed.

## 1. Introduction

Ocean color sensors based on polar-orbiting satellite platforms have been providing invaluable synoptic information about the optical and biogeochemical properties of the surface ocean since the 1978 launch of the Coastal Zone Color Scanner [1]. Contemporary ocean-sensing radiometers (e.g., VIIRS and OLCI) are specifically engineered to detect the comparatively weak radiance signal emerging from the surface ocean [2]. Removal of the intervening atmospheric signal (that may account for more than 90% of the total visible band radiant energy received) as well as radiometric sensor calibration requires continuing verification and validation efforts [3,4]. Provided these endeavors are successful, these sensors may indeed provide reasonable estimates of measurable sea surface quantities [5,6]. 

Most of these satellite-based sensors, however, (with the notable exception of the Geostationary Ocean Color Imager, GOCI [7]) are subject to the temporal constraint that a local area of ocean may be observed once per solar day (or perhaps less frequently due to orbital geometry). The presence of clouds may further render some ocean areas unobserved for days to weeks. Nonetheless, coverage is often sufficient to resolve the physical-biological interactions occurring within the oceanic mesoscale, that is, surface ocean circulation features on the spatial scale of tens to hundreds of kilometers across and persisting for several weeks to months. For example, the mesoscale eddies (large centers of cyclonic or anticyclonic ocean circulation) are often detected in satellite radiometer data, particularly near highly energetic western boundary currents [8]. 

Although the mesoscale remains an area of ongoing research, oceanographers are now devoting increasing attention to processes occurring on smaller space-time scales, a domain referred to as the “submesoscale” [9]. The submesoscale may be regarded as oceanic processes occurring on spatial scales of a few kilometers and smaller, and temporal scales of hours to a few days. Yet a critical aspect of the submesoscale paradigm is the dynamical ocean circulation: the submesoscale involves vertical movements of water several orders of magnitude more rapid than those typical of the larger mesoscale ocean circulation [10]. Thus, these smaller scale movements may have a cumulatively large impact on global ocean processes, such as the biogeochemical cycling of elements [9,11]. 

Comprehensive observation of the ocean submesoscale from space requires sub-kilometer image resolution as well as a very high frequency data acquisition. These requirements virtually mandate a geostationary or geosynchronous satellite orbit [12,13]. The Geostationary Operational Environmental Satellite—R Series (GOES-R) meet these requirements, however, the Advanced Baseline Imager (ABI) sensor was not designed for ocean color applications [14]. ABI lacks the visible spectral resolution (there are only two, broad visible bands centered at 470 and 640 nm) and the dynamic range and signal sensitivities that are typical of contemporary ocean color imagers. Nevertheless, the GOES-ABI information may be repurposed to observe coastal ocean processes if the ABI visible band data are convolved with data obtained from dedicated ocean-viewing radiometers [15]. 

This general concept of polar-orbiting and geostationary satellite synergy has been employed previously to convolve Moderate Resolution Imaging Spectroradiometer (MODIS; NASA-Aqua satellite) multispectral data with Spinning Enhanced Visible and Infrared Imager (SEVIRI; geostationary) data in order to observe coastal turbidity at very high temporal frequency [16], but these efforts were restricted to the single SEVIRI visible band (red-635 nm). The additional blue visible band of GOES-ABI has motivated efforts to perform true color image estimation from GOES-ABI data using a variety of methods to generate a synthetic green band [17]. In many cases look-up tables or statistical regression factors are generated by utilizing complimentary multispectral data from MODIS, VIIRS, or other sensors [18,19,20]. The “Geocolor” product available from NOAA GOES image viewer (https://www.star.nesdis.noaa.gov/GOES/) uses a look-up table built from the Advanced Himawari Imagers (AHI: includes a green, 510 nm band [21]) on the geostationary, Asia-Pacific viewing Himawari-8 and-9 satellites [22,23]. However, these synthetic GOES-R true color products are primarily focused on atmospheric/meteorological or terrestrial applications. The ocean color reflectance signal is very different in magnitude and tendencies from these other signals, and it requires a dedicated methodology. 

In this paper, we present a method for performing polar-orbiting-to-geostationary sensor data convolution and true color image estimation for GOES-R series ABI data that is specific to ocean color, i.e., the radiant signal emerging from the ocean’s surface. The new method, Chromatic Domain Mapping (CDM), is applied to GOES-R ABI scenes in the Gulf of Mexico. Section 2 provides an explanation of colorimetry as applied to ocean remote sensing and the CDM technique. Section 3 presents a series of example CDM applications: (Section 3.1) the coastal ocean response to Hurricane Michael, (Section 3.2) local expansion of the Suwannee River plume over the West Florida continental shelf and potential resuspended sediment settling, and (Section 3.3) marine frontal boundary movement/subduction following atmospheric cold front passage. These examples demonstrate the unprecedented detail and temporal resolution available using the GOES-ABI data in conjunction with the CDM methodology described herein. 

## 2. Materials and Methods

Satellite data were obtained from three sensors: (1) the Ocean and Land Colour Imager (OLCI) on board the Sentinel-3A satellite; (2) the Visible Infrared Imaging Radiometer Suite (VIIRS) on the Suomi-National Polar-Orbiting Partnership (NPP) satellite; and (3) the Advanced Baseline Imager (ABI) on the Geostationary Operational Environmental Satellite (GOES)—R series (East). 

### 2.1. True Color Reconstruction from Visible-Band Satellite Products

For sensors (1) and (2) the Level 1 data were processed using the Naval Research Laboratory’s (NRLs) Automated Optical Processing System (AOPS) [24]. The software system performs the appropriate atmospheric correction and produces Remotely-sensed reflectance (*Rrs*, sr^−1^) at 300 m horizontal resolution (OLCI) and 750 m resolution (VIIRS). *Rrs* from 7 selected OLCI visible bands (412, 443, 490, 560, 665, 671, and 681 nm) and 5 VIIRS visible bands (412, 445, 488, 555, and 672 nm) were subjected to a band-centered, cubic spline interpolation procedure in order to construct an estimate of the hyperspectral *Rrs* signature (Δλ = 1 nm, 400–700 nm) for each valid ocean pixel. 

The AOPS processing conforms to standard NASA protocols [25], and the *Rrs* products are based on the normalized water-leaving radiances: *Rrs* = *nLw*/*F*_0_, where *F*_0_ is the mean extraterrestrial solar irradiance. *Rrs* (sr^−1^) and *nLw* (mW·cm^−2^·um^−1^·sr^−1^) are the primary geophysical products used in ocean color remote sensing because their variance is presumed to be dominated by changes in the optical properties of the surface ocean [26] that are, in turn, influenced by various biogeochemical processes [27]. *Rrs* may be multiplied by π to give the dimensionless water-leaving reflectance [*ρ_w_*] [28].

Where atmospheric (aerosol) correction proves difficult, [*ρ_w_*] may be replaced by [“rho_s”; *ρ*_s_], which is the estimated surface reflectance that has not been corrected for aerosol atmospheric contamination or aerosol-Rayleigh interactions. This product is corrected for strictly Rayleigh contamination, atmospheric gas transmittances, and solar zenith angle; it is designated as quasi-surface reflectance in NASA product documentation [29]. 

Quantitative color reconstruction from these reflectance products [*ρ_w_*, *ρ_s_*] is based on the method described in Wernand at al. [30,31] and is more generally the method used for standard colorimetric analysis of hyperspectral reflectance (or transmittance) data [32]. This is a deliberate departure from much of the “true color” satellite imagery that appears in oceanographic literature and elsewhere. In many of these cases, three satellite radiometer channels are selected and arbitrarily scaled (to a range of 0–255) to construct a three-channel red-green-blue (RGB) color image. It is difficult to quantitively reproduce this color-rendering method because the scaling for each channel is deliberately arbitrary. Herein, the interpolated radiant spectra (from either remotely-sensed reflectance or quasi-surface reflectance) is integrated (via Reimann sum approximation) with the CIE 1931 standard color matching functions [33]:

(1) $$Xr=\int_{400}^{700}\rho_{w\left(s\right)}\left(\lambda \right)\;\;X_{CIE\;}\left(\lambda \right)\;D_{65\;}\left(\lambda \right)d\lambda$$

(2) $$Yr=\int_{400}^{700}\rho_{w\left(s\right)}\left(\lambda \right)\;\;Y_{CIE\;}\left(\lambda \right)\;D_{65\;}\left(\lambda \right)d\lambda$$

(3) $$Zr=\int_{400}^{700}\rho_{w\left(s\right)}\left(\lambda \right)\;Z_{CIE\;}\left(\lambda \right)\;D_{65\;}\left(\lambda \right)d\lambda$$

The subscript (*r*) indicates raw integrals (tristimulus values) across the CIE 1931 tristimulus functions (2° Field-of-View). The three tristimulus functions (*X_CIE_*, *Y_CIE_*, and *Z_CIE_*) begin at 360 and extend to 780 nm, however, the functions are truncated herein (400–700 nm) and the bulk of the tristimulus function sensitivities are within this restricted spectral range. The *D*_65_ term is the standard illuminant for daylight, outdoor conditions. Reflectance colorimetry computations are usually performed with a specified standard illuminant, such as *D*_65_. Other choices are permissible so long as the tristimulus functions and illuminance standards are specified in the color computation and applied consistently. Taken together, the ocean color product and the colorimetry computation results in raw tristimulus values that would correspond to the color perception of an observer looking directly down into the water (no surface perturbation). 

Unlike standard reflectance scenes familiar to photographers and other color science applications, the true water-leaving reflectance signal from the surface ocean is very small. For example, a typical ocean remote sensing *ρ_w_* value of ~0.003–0.01 is well below a standard middle grey value of 0.18 (middle grey is perceptually half way between black and white). That is why a spectrally uniform brightness standard (*B_ref_*) in the brightness equation must be specifically designated for ocean remote sensing colorimetry applications:

(4) $$Ys=\int_{400}^{700}\;B_{ref}Y_{CIE\;}\left(\lambda \right)\;D_{65\;}\left(\lambda \right)d\lambda$$

and

(5) $$X=\frac{Xr}{Ys},\;Y=\frac{Yr}{Ys},\;Z=\frac{Zr}{Ys}$$

The standard *X*, *Y*, and *Z* (red, green, and blue) tristimulus values may also be expressed in chromaticity coordinate space by defining the normalized *x*, *y*, and *z* chromaticity values:

(6) $$x=\frac{X}{\left(X+Y+Z\right)},\;y=\frac{Y}{\left(X+Y+Z\right)},\;z=\frac{Z}{\left(X+Y+Z\right)}$$

Note that chromaticity coordinates do not depend on relative brightness (reflectance magnitude), but are instead dependent upon the shape of the radiant power distribution. In a typical chromaticity diagram, *x* and *y* are displayed and *z* is omitted (since only *x* and *y* are required to identify a unique position in chromaticity space). However, for conversion to other color spaces wherein brightness is required, at least *Y* of the tristimulus primaries (*X*, *Y*, and *Z*; note chromaticity coordinates are by convention lower case *x*, *y*, and *z*) will be needed for additional computation. Chromaticity coordinates (*CIE* *xyY*) were converted to standard RGB for display as JPEG images following conventional color space conversion methods [34]. As long as the brightness reference is indicated, then (1) the color-rendering method is reproducible, and (2) the chromaticity of natural waters (*x*, *y*, and *z* coordinates) may be examined independently of the brightness standard selected. 

**Figure 1:**
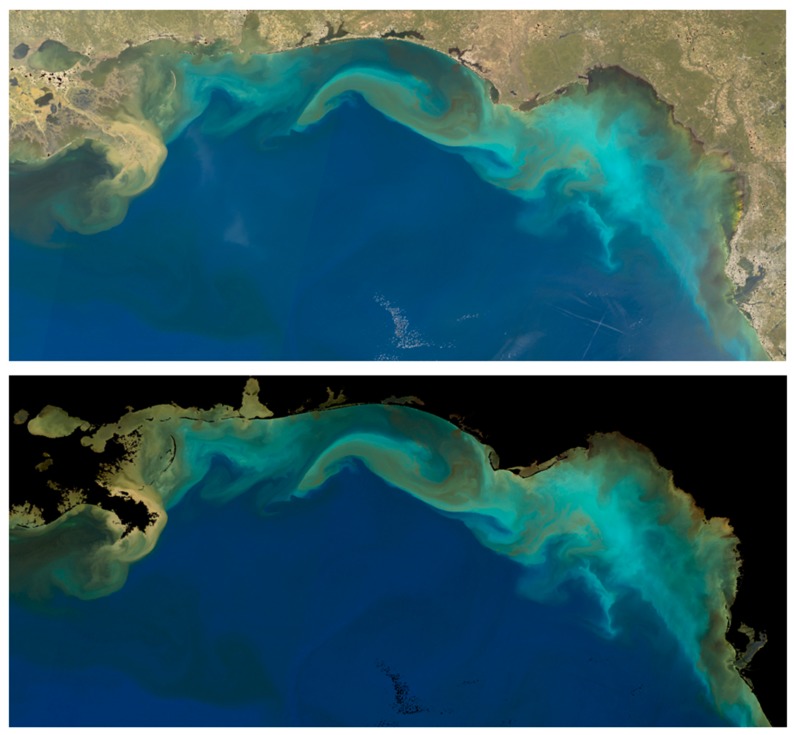
OLCI true color reconstruction: (**top**) true color based on quasi-surface reflectance and (**bottom**) remotely-sensed reflectance (*Rrs*), reference brightness for both scenes is 15%. Clouds and land are not removed from the quasi-surface reflectance product.

An example of the results of the aforementioned true color reconstruction from OLCI reflectance data is shown in Figure 1. The [*ρ_s_*] product (top) includes land and clouds, and these features are masked from the [*ρ_w_*] product (bottom). Despite the lack of a thorough atmospheric correction (top), the ocean features of the two images have very similar color characteristics. This suggests that dominant color characteristics of marine waters may, in some cases, be estimated from reflectance data even where aerosol correction has not been performed. At a minimum, sharp gradients in ocean true color that are very often indicative of frontal boundaries may be located. 

### 2.2. GOES-ABI Processing 

GOES-ABI (East) has two broad bands in the visible, one centered at 470 nm and one at 640 nm. The GOES ABI data for the study was retrieved from the Comprehensive Large Array-Data Stewardship System (CLASS) website (https://www.avl.class.noaa.gov/saa/products/welcome). Data selection parameters for the first 3 channels of the ABI L1B radiance datatype of GOES 16 for the CONUS extent were used to search for GOES data over the days of interest. The GOES Level 1B (L1B) data files were integrated and reformatted into one NRL systems-compliant L1B file to conform to the input format required by the AOPS program. The NRL-compliant L1B files were batch processed by AOPS to reproduce the “quasi-surface reflectance” product [*ρ_s_*] at the 470 and 640 GOES-ABI bands. This allows for direct comparison to OLCI and VIIRS products without aerosol correction. 

### 2.3. Chromatic Domain Mapping 

Chromatic Domain Mapping (CDM) is a procedure to quantify how the *X*, *Y*, and *Z* tristimulus primaries are related to one another in a reference color image, and then use this information to determine the most likely estimator of one primary in a target image where that primary is corrupt, incomplete, or missing. This method is tractable for images based, primarily, on the ocean color signal, i.e., the water-leaving radiance (*L_w_*). This is because the spectral shape of the *L_w_* signal is largely a function of the spectral Inherent Optical Properties (IOPs) of the surface ocean [35]. These variable IOPs, in turn, are due to the concentration of various optically-active substances in marine waters, e.g., chromophoric dissolved organic matter (CDOM), phytoplankton pigments, and suspended organic and inorganic particles. Whereas the relationships between the concentration of these constituents and their respective spectral optical properties is very complex, the spectral shapes of the resultant reflectance signals tend to vary in recurring patterns, a feature that has long been exploited in the development of ocean color inversion algorithms (e.g., reference [36]). 

**Figure 2:**
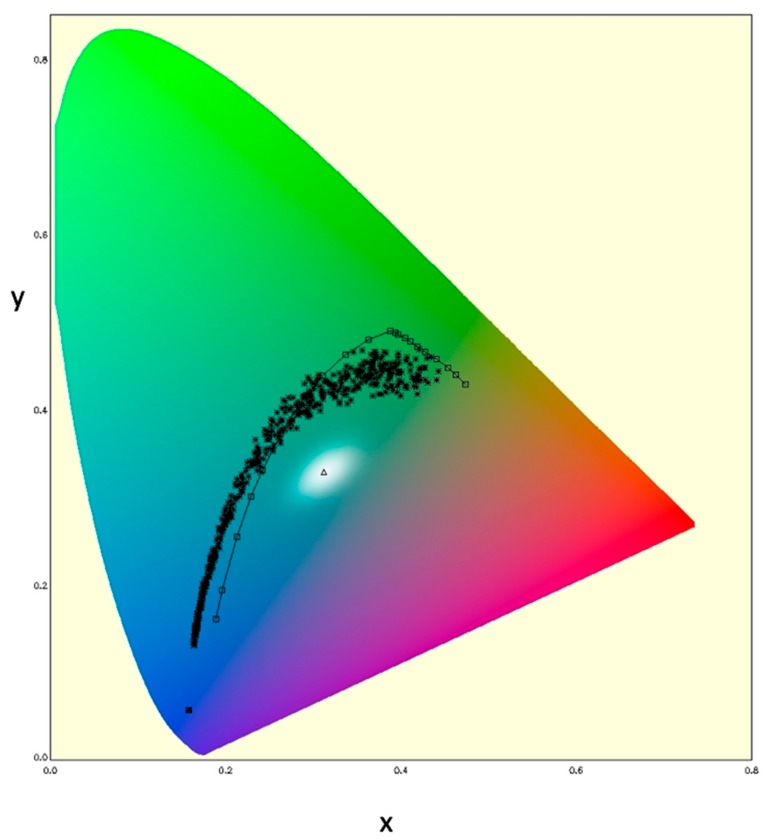
Chromaticity diagram showing the IOCCG reference spectra (*n* = 500) in chromaticity space (*). The squares and line are the FU scale color reconstructions from Wernand et al. [30].

These spectral patterns for marine waters may be very succinctly summarized via colorimetric analysis. For example, the International Ocean Colour-Coordinating Group (IOCCG) has established 500 reference *Rrs* spectra that are representative of marine waters [37]. These spectra were used in Equations (1)–(6) (*ρ_w_* = *Rrs* π); and the results displayed in chromaticity coordinate space (Figure 2). A reference point based on the IOPs of pure seawater [38] and a simplified computation of the resultant *Rrs* hyperspectral signal [26] is shown as well. Pure seawater (far lower left) and very clear ocean waters occupy the lower left domain of the chromaticity diagram (small *x* and *y* values, indicating a dominance of blue light, *z*). Generally, increasing water turbidity follows a characteristic arc, first increasing predominantly along the *y* axis (increasing relative amount of green light) and then increasing more along the *x* axis (increasing relative amount of red light). The term “turbidity” is used here simply to refer to an overall increase in absorbing and scattering optical constituents for marine waters. Also shown in the diagram are the Forel-Ule (FU) color comparator scale chromaticity coordinates, as given in Wernand et al. [30]. These FU values follow a similar trend, increasing first in y then in *x* coordinates with increasing turbidity; there is even an inflection towards diminishing *y* values at the right-side end of the FU spectrum (Figure 2). 

The overall philosophy of the CDM method follows from this diagram: hyperspectral signals from marine waters occupy very specific regions of chromaticity space and once the location is estimated for a given sample, other properties may then be inferred. For the GOES-ABI data, it is presumed that the 470 band is a suitable estimator of the blue primary, *Z*, and the 470 band and 640 bands are (combined) likewise estimators of the red primary, *X* (the red CIE 1931 tristimulus function includes some sensitivity in the blue spectral region). The *Y* primary is missing; GOES-ABI does not have a green band. The problem posed: given an estimate of *X* and *Z*, what is the most likely estimate of *Y*? To initially remove variations due to changes in brightness (signal magnitude), it is prudent to first examine the behavior of a reference color image in chromaticity space. However, since the *Y* primary is unknown, we reduce the problem further to examine the relationship between simple tristimulus value ratios. 

**Figure 3:**
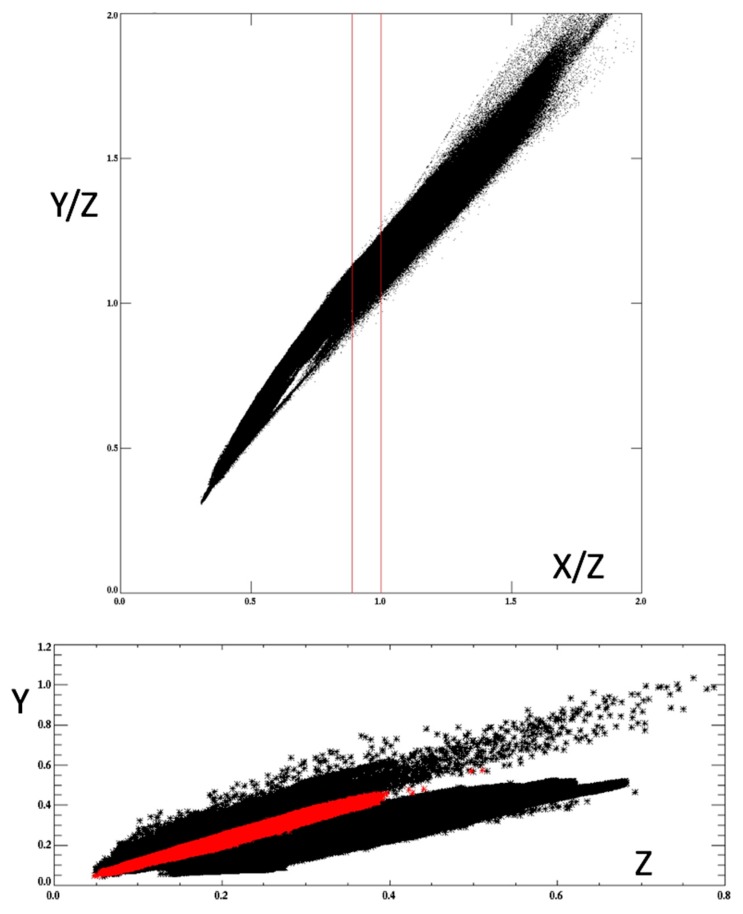
Tristimulus primary data extracted from OLCI rho-s (Figure 1, **bottom**); (**top**) primary ratios; (**bottom**) *Y* versus *Z* primary—the red indicates a restricted range for the primary ratio *X*/*Z*.

For example, the OLCI color image data in Figure 1 (top, based on *ρ*_s_) is shown in Figure 3 to demonstrate the quasi-linear relationship between the *X*/*Z* ratio (a quantity that may be estimated from GOES-ABI data) and *Y*/*Z* ratio (the unknown). This reference relationship can be exploited by defining the minimum and maximum values of *Y* for a given value of *Z* and *X*. The ratio *X*/*Z*, rather than the absolute *X* and *Z* values, is initially critical because this ratio narrows the possible chromatic domain down to a restricted range of optical water types. Recall the chromaticity diagram (Figure 2) for the reference spectra: clear waters are blue light dominant, and very turbid waters are red light dominant. Similarly, The *X*/*Z* ratio can be conceptualized as a bulk indicator of water turbidity ranging from clear marine waters (low red to blue primary ratio) to more turbid (higher red to blue primary ratio).

To determine the absolute magnitude of *Y* in the target image that is within the minimum and maximum bounds established by the reference image, we then examine the linear relationships between *X* (or *Z*) and *Y* over a very restricted range of *X*/*Z* values. For example, there is significant scatter and divergence when all *Y* versus *Z* values are plotted in the reference image (*r*^2^ = 0.46; Figure 3 (bottom)). Over a restricted range of *X*/*Z* values, however, there is a much more linear relationship (*r*^2^ = 0.99) that will conform to simple linear regression techniques

**Figure 4:**
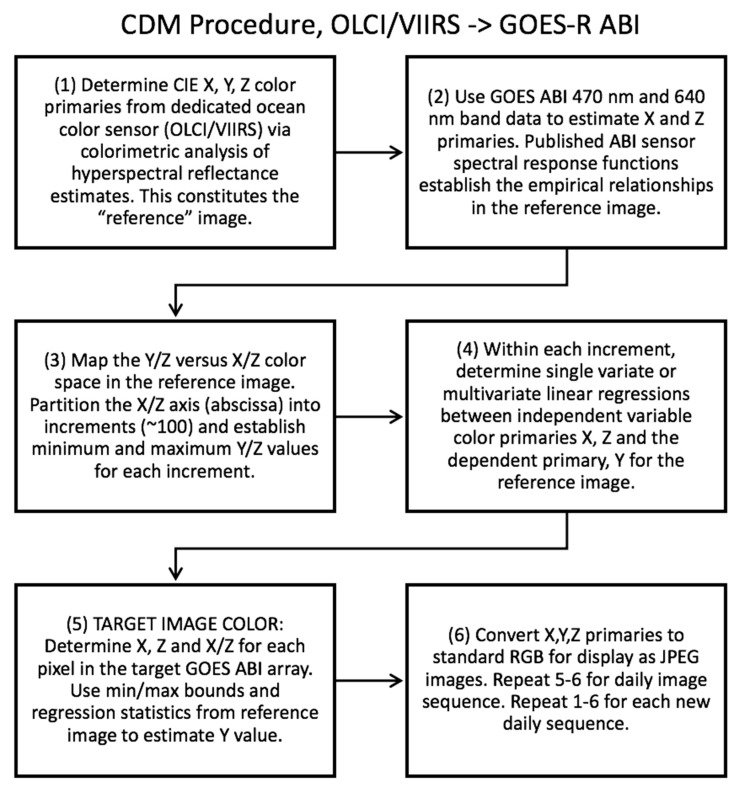
Flow diagram of CDM procedure for generating true color image sequences from GOES-ABI data.

Chromatic Domain Mapping is the process of quantifying these relationships in the reference image and then determining the most likely missing or corrupt primary in the target image. In theory, these relationships could be established from reference *Rrs* spectra for marine waters. However, we have not yet established a consistent method to remove aerosol contamination from GOES-ABI blue and red band data. Thus, the reference image is the non-aerosol corrected surface reflectance product, with land and clouds (as much as possible) eliminated from the reference image for the CDM application. A flow diagram of the CDM procedure for the specific application of convolving OLCI or VIIRS data to the GOES-R ABI image sequences is provided in Figure 4. 

**Figure 5:**
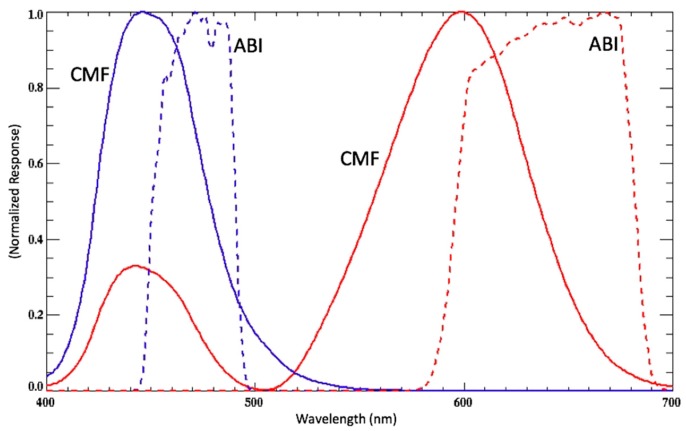
Blue and red color matching functions (CMF-solid lines) and GOES-ABI spectral response functions (dashed lines).

Step 1 (Figure 4), the colorimetry analysis of ocean color sensor reflectance, is described in detail in the preceding Section 2.1. and Equations (1)–(6). In step 2 (Figure 4) it is important to clarify that although GOES-ABI only has two bands in the visible, these bands are very broad and thus the ABI spectral response covers significant portions of the visible. These features make the ABI well-suited to estimate red and blue color primaries (*X* and *Z*) since the color matching functions (CIE 1931) are also very broad (Figure 5). When estimating color properties from satellite visible band data, one must not only consider the total number of bands in the visible range but also the spectral shape (band width) of the spectral response functions. In the same manner that a hyperspectral reconstruction from multispectral (narrow band) sensor data can be integrated with the color matching functions, these hyperspectral reflectances can also be integrated with the ABI spectral response functions (Figure 5). There is sufficient overlap between color matching functions (for *X* and *Z*) and GOES ABI spectral responses that linear relationships between them may be determined for a reference image (Step 2; Figure 4). Once the *X* and *Z* values are estimated, the remaining task is to estimate the missing *Y* value, and this is done using the *X*, *Y*, and *Z* primary relationships established in the reference image (Step 3). 

We initially applied 100 increments to the reference *X*/*Z* ratio values (Step 3; Figure 4) as this resulted in at least ~100 pixels within each increment given an OLCI swath width of ~1270 km, a selected scene (granule) height of comparable distance, and a pixel resolution of 300 m. More increments will reduce the number of pixels, and we presumed that below ~30 (based strictly on a common statistical rule-of-thumb estimate) the regressions would become less accurate. Introducing fewer *X*/*Z* increments (and larger sample sizes), however, will cause the primary relationships within each increment become less linear (as shown in Figure 3 (bottom)). Hence there is an inherent compromise to be made between sample size and linearity. Further refinement of the method will determine a more robust method of total increment partition. Within each increment, the minimum and maximum *Y*/*Z* values are simply determined by the total range of pixels in that increment. If a spurious pixel with an abnormally high or low *Y*/*Z* value is present, this range will not be accurate and the linear regressions (Step 4) may be unduly biased. Thus, some manual quality control is required when establishing the reference pixels (Figure 3 (top)). 

Steps 5 and 6 are the rendering processes for converting the GOES ABI data into color image sequences. Due to the aerosol contamination that remains in the reference image at this time (and until GOES ABI data are aerosol corrected), it is recommended that the reference image from OLCI or VIIRS be in close in time and space as possible to the GOES ABI image sequence. More work is needed to determine how the latency of the reference image impacts the GOES ABI color enhancement. 

The *X*/*Z* ratio determination narrows the likely optical water type along a spectrum from turbid (higher *X*/*Z* ratios) to very clear (lower *X*/*Z* ratios). Once this restriction of the data occurs then (1) the range of possible *Y* values is restricted, and (2) relationships between *X*, *Y*, and *Z* primaries are much more amenable to simple linear statistics and the true color reconstruction maintains fidelity to the reference color image. A separable and distinct issue is then to demonstrate what advantage the true color rendering offers that examination of a single ABI channel (470 or 640 nm) does not. This issue is explored in the following series of example applications. 

## 3. Results

### 3.1. CDM Product Verification and 13 October 2018 GOES-ABI Sequence

The OLCI-based color image [*ρ_s_*] in Figure 1 (top) is designated as the reference image and the land mask from the [*ρ_w_*] product (Figure 1 (bottom)) is applied to remove land surface reflectance. The CDM software (written in Interactive Data Language) parses the reference image *X*/*Z* ratio values into 100 increments and then performs a chi-square minimization linear fit to the *X* (independent) and *Y* (dependent) tristimulus values within each increment. Linear fitting for *Z* (independent) or multivariate (*Z* and *X*) are also performed with the option of selecting the superior statistical performer. The increments and linear fit statistics are retained for application to the target image. 

**Figure 6:**
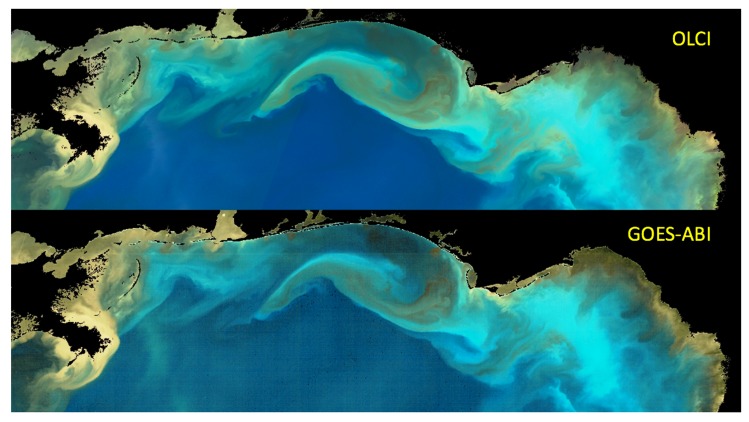
(**top**) OLCI (Sentinel 3A) true color reconstruction, quasi-surface reflectance, 15:55 UTC; (**bottom**) GOES-ABI, CDM-enhanced image 15:02 UTC. See also Video S1.

The technique was first applied to the GOES-ABI (East) image sequence obtained on 13 October 2018, during the ocean response to the passage of Hurricane Michael through the Gulf of Mexico. The enhanced GOES-ABI image sequence faithfully reproduces the dominant color patterns detected in the OLCI true color reconstruction (Figure 6). The mud-brown of the Mississippi River Delta (MRD) effluent is a particularly distinctive turbidity signal that is, in color space, very distinct from the shelf sediment resuspension color signal [39,40]. Accordingly, the bright turquoise hues evident in the color reconstructions near the Florida Big-Bend region are likely indicative of shelf sediment resuspension processes. The darker brown highlight colors also evident there suggest elevated chlorophyll concentrations, due to either phytoplankton stimulated by nutrients within the resuspended sediment plumes or due to the direct resuspension of benthic algae [41]. 

**Figure 7:**
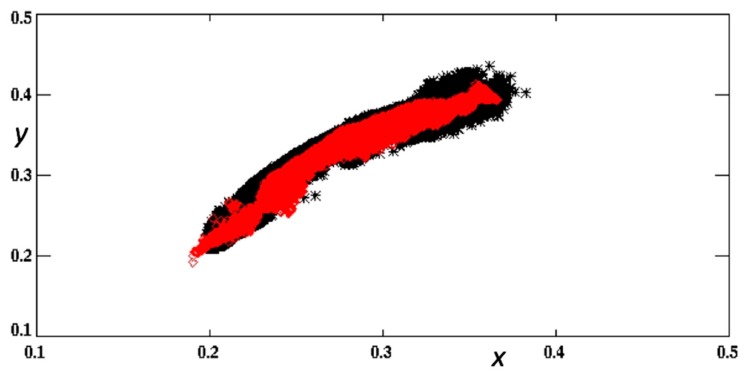
The color information (as shown in Figure 6) from OLCI (black) and color-enhanced GOES-ABI (red) is displayed in the *x*,*y* chromaticity plane.

Verification of the color product was performed by remapping the OLCI color data to the GOES-ABI product grid, and then directly comparing color properties on a pixel-by-pixel basis. The results are shown in the (*x*,*y*) chromaticity plane in Figure 7. The critical features of the CDM processes are that (1) fidelity to the chromatic domain of the reference image is maintained, and (2) spurious true color discontinuities are avoided. The average OLCI versus GOES-ABI Euclidean distance in the *x*,*y* chromaticity plane was 0.03 (*n* = 343,053). The GOES-ABI color reconstruction generally conforms to the shape and boundaries of the OLCI data in the chromaticity plane. Moreover, the color reconstruction (Figure 6) does not introduce spurious or obvious color discontinuities that would potentially result from a look-up-table approach to either the color primaries or the underlying reflectance values. This is an important point because true color discontinuities (based solely on the water-leaving radiance signals) in coastal areas are often indicative of water mass frontal boundaries. Spurious artifacts in the color field would render false identification of such boundaries. 

Setting aside color-enhancement verification, another salient point is the value of the true color reconstruction as opposed to examining a single GOES-ABI band reflectance value. For example, Nechad et al. [42] provide evidence that a single red channel may be used to determine total suspended material concentration in coastal areas. Generally, true color is superior to a single channel because (1) the color signal is an integration across the entire visible spectrum, and (2) true color gradients may be detected even if the optical properties of the underlying constituents are not well known. Sulfide eruptions off the coast of Namibia [43], anomalous “black water” events on the West Florida Shelf [44], and surface carbonate precipitation [45], are but a few examples of natural events that are detected in satellite visible band imagery that do not conform to the standard suspended sediment or (chlorophyll-*a*) phytoplankton bloom paradigm. 

**Figure 8:**
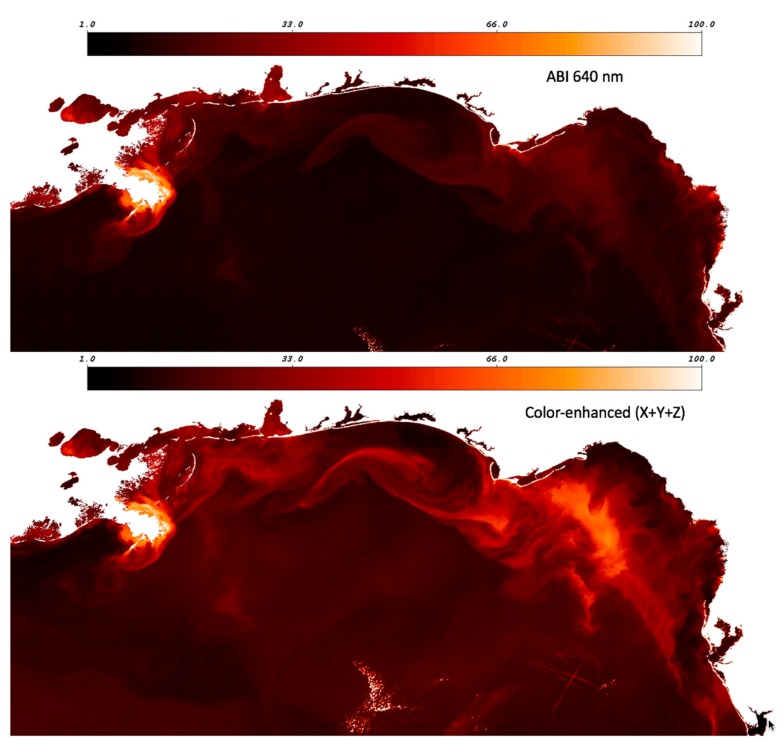
GOES 640 nm band surface reflectance, northern Gulf of Mexico, normalized values (**top**), and (**bottom**) GOES-ABI enhanced color sum of primaries (*X*+*Y*+*Z*), normalized values.

This particular scene in the Gulf of Mexico is a good example of this concept. The single red channel GOES-ABI reflectance scene does indeed show elevated values near the Mississippi River Delta, where suspended sediment concentrations are presumably quite high (Figure 8). However, far less red-band signal strength or spatial detail is revealed farther to the east towards Florida (Figure 8), and this may be demonstrated via a comparison to the simple sum of the color primaries (*X*+*Y*+*Z*). As shown in Jolliff et al. [40], the turbidity associated with resuspended shelf sediments in the northern Gulf of Mexico, either along the ocean bottom in the nepheloid layer or ventilating to the surface following storm events, has a unique spectral signature that is shifted towards the blue end of the visible spectrum. It is nonetheless extremely turbid (beam attenuation values at 532 nm approaching 10 m^−1^). Jolliff et al. [39] identified such a sediment resuspension feature in Hyperspectral Imager for the Coastal Ocean (HICO) data, and found the spectral peak at ~486–515 nm, outside the spectral responsiveness of the GOES-ABI red band (as shown in Figure 5). 

Thus, the enhanced-color GOES images largely preserve the optical richness of the ocean color scene whilst the image sequences (see Supplementary Materials Video S1) reveal unprecedented information about the kinematics of the coastal ocean. The presumed sediment and algae plumes southwest of Cape San Blas are observed here to rotate anticyclonically. To the left side of the image, the active discharge of freshwater from the Mississippi River Delta (MRD), and particularly the Southwest Pass, is prominent. The rapid movement of the frontal boundary between the MRD effluent and the pelagic Gulf of Mexico waters is evident there as well. The movement of smaller estuarine plumes can be seen south of Mobile Bay, Mississippi Sound, and Choctawhatchee Bay. The specific optical composition (for example, IOP values, chlorophyll concentrations) may be estimated by the OLCI/VIIRS data, but the coincident GOES-R enhanced-color image sequence data may then determine the minutes to hourly movements of these features.

Whereas the OLCI (reference image) swath is limited to 1270 km, the full hemispheric disk is available to GOES-R. The same color map may be applied to a wider area of the Gulf of Mexico (Supplementary Materials Video S2) that extends beyond the immediate OLCI sensor swath. Due caution must be exercised when extending beyond the reference swath since aerosol properties, that remain uncorrected, may change. In the expanded view, additional sediment resuspension may be occurring south of the Atchafalaya River plume.

### 3.2. 10 January 2019 GOES-ABI Sequence

In contrast to the turbidity of the Mississippi and Atchafalaya River plumes, an image sequence obtained for the West Florida Shelf immediately following the passage of an atmospheric frontal system bringing cold, dry air masses (also known as a cold-air outbreak (CAO) event [46,47]) potentially reveals two different processes: (1) the movement and expansion of the much darker Suwannee River plume; and (2) potential settling of resuspended sediments farther south along the Saint Petersburg, Florida peninsula.

**Figure 9:**
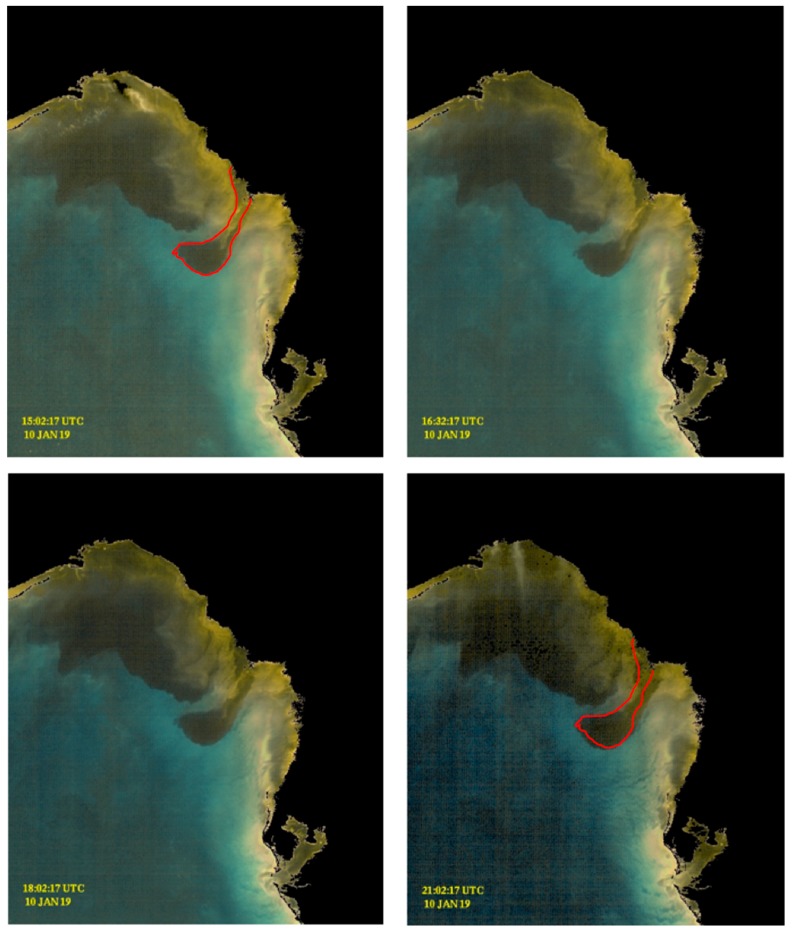
Enhanced GOES-ABI sequence for 10 January 2019, on the West Florida Shelf (see also Supplementary Materials Video S3). Prominent features include the expansion of the Suwannee River Plume (SRP) and potential settling of shelf sediments. The outline of the estimated initial position of the SRP is shown in the first and last color-enhanced GOES image frame.

The Suwannee River is considered a “black-water” river due to the very high concentration of chromophoric dissolved organic matter (CDOM) [48,49], as are the smaller rivers in the Florida Big Bend region. Rainfall associated with the passage of atmospheric frontal systems elevated the Suwannee River discharge (as well as other rivers in the region), and in this image sequence the discolored, black waters emanating from the Suwannee River estuary appear delineated from other waters and are conspicuous. The southern edge of the plume appears to propagate southward ~1.5 km over a 6-h period (Figure 9; Supplementary Materials Video S3). 

USGS data indicate instantaneous discharge rates of approximately 285 m^3^·s^−1^ during the previous 24-h period [50], well above the long-term average for this time of year. This discharge rate would suggest, in addition to the potential advection of the plume waters, a plume expansion of ~12–24 km^2^·d^−1^ while neglecting evaporation/precipitation and mixing with seawater. This would translate into the discoloration of ~50–100 additional image pixels. This appears to underestimate the apparent rate of plume expansion in Figure 9 (lower right), but it is nonetheless reasonable given the remaining uncertainties. The key point is that the GOES-ABI color-enhanced sequence now provides high resolution temporal information about coastal features of interest that have long been examined in temporally “static” satellite “ocean color” images. 

Just to the south of the Suwannee River plume and towards the Florida coast, the brighter discoloration may be consistent with shelf sediment resuspension. Strong northerly winds are a typical feature of northern Gulf of Mexico CAO events [51], and the WFS circulation response would be a strong upwelling circulation mode via bottom and surface frictional boundary layers [52]. This is conceivably enough stimulus (bottom stress) to resuspend nearshore shelf sediments. However, as the image sequence progresses the northerly winds are simultaneously decelerating (NOAA Buoy meteorological data not shown). Apparent disruptions of the brightness pattern evident in the GOES-ABI enhanced true color sequence may be indicative of the corresponding settling of resuspended shelf sediment (Figure 9, Supplementary Materials Video S3). 

### 3.3. 24 January 2019 GOES-ABI Sequence

**Figure 10:**
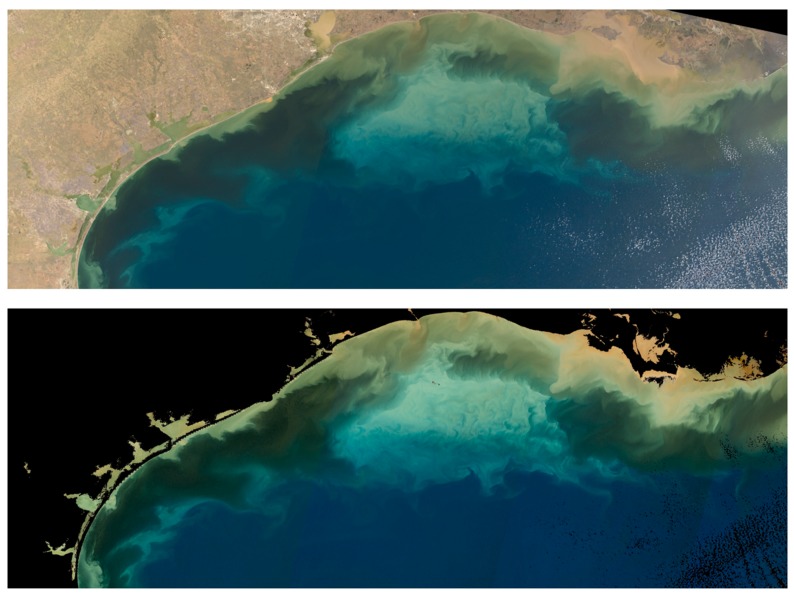
OLCI (Sentinel 3A) true color reconstruction 24 January 19, 16:29 UTC, central and northwestern Gulf of Mexico (top *ρ_s_*; bottom *ρ_w_*). Note there is some atmospheric correction (aerosol) failure in the bottom image.

A prominent example of submesoscale processes occurs in the northern Gulf of Mexico following a CAO event later that same month. The turbidity and low-salinity plume from the Atchafalaya River discharge appears to extend out unto the shelf and this is clearly depicted in true color reconstructions based on OLCI (Sentinel 3A) data (Figure 10). The apparent brightness “cloud” in the center of the image is likely to be resuspended shelf sediments. The darker, brown-water turbidity features farther north and east towards Atchafalaya Bay are indicative of river sediment loads combined with very high levels of CDOM [53]. This true color contrast is critical: shelf water resuspension and turbidity versus river plume turbidity is likely indicative of a density gradient, due to both salinity and temperature differences. The apparent movement of the boundary between these true color features is thus also indicative of frontal (density) boundary dynamics. 

**Figure 11:**
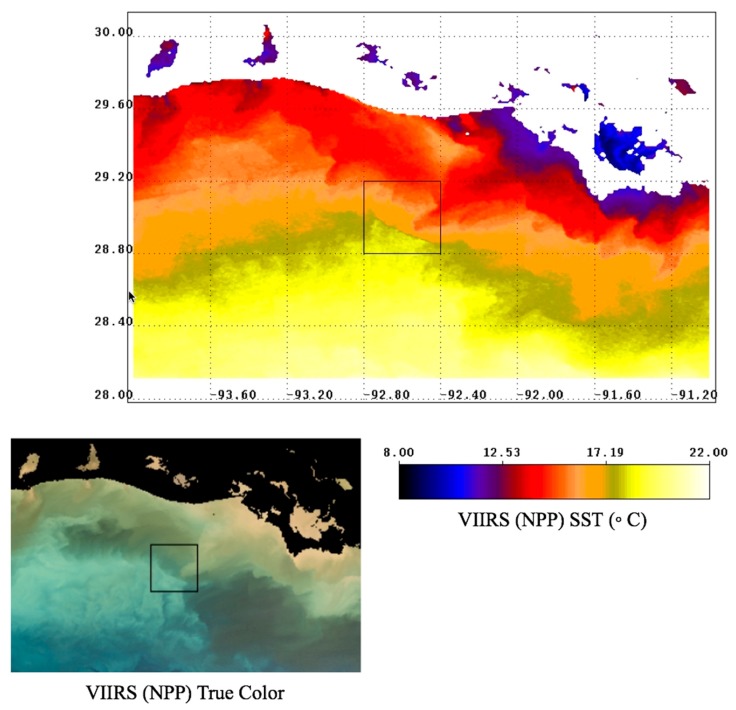
VIIRS (NPP) SST and true color (lower left) for 24 January 19, 1900 UTC, north-central Gulf of Mexico.

The VIIRS (NPP) sensor obtained color information ~2.5 h later (1900 UTC) and there was apparent movement of the features identified in initial OLCI scene (Figure 11; area bounded by 92.4°–92.8° W and 28.8°–29.2° N). The VIIRS SST indicates the color gradient is also a temperature gradient (see inset Figure 11). The temperature gradient supports the inference that the lighter discoloration is indicative of marine turbidity whereas the darker discoloration is the river plume-influenced turbidity (lower salinity). As explained further below, the atmospheric CAO event will much more effectively reduce the sea surface temperature of shallower shelf areas or shelf areas where a strong halocline is present. 

**Figure 12:**
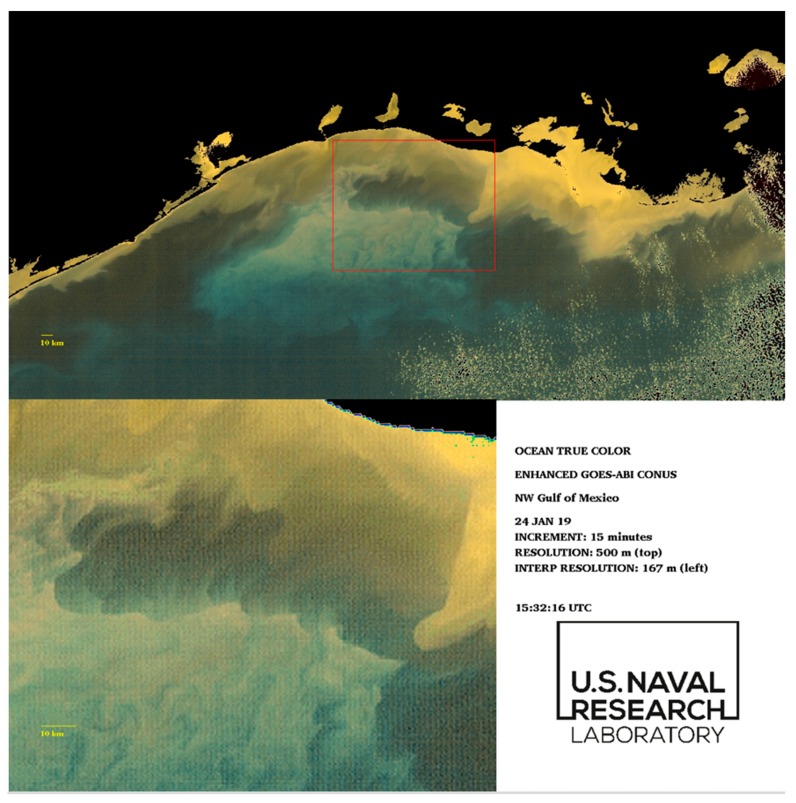
Initial frame of enhanced GOES-ABI sequence for 24 January 2019, northern Gulf of Mexico. The red box (**top**) indicates frontal boundary zones; (**bottom**) the bottom panel is an interpolated increase in image resolution by a factor of 3. The full image sequence is available in Supplementary Materials Video S4.

**Figure 13:**
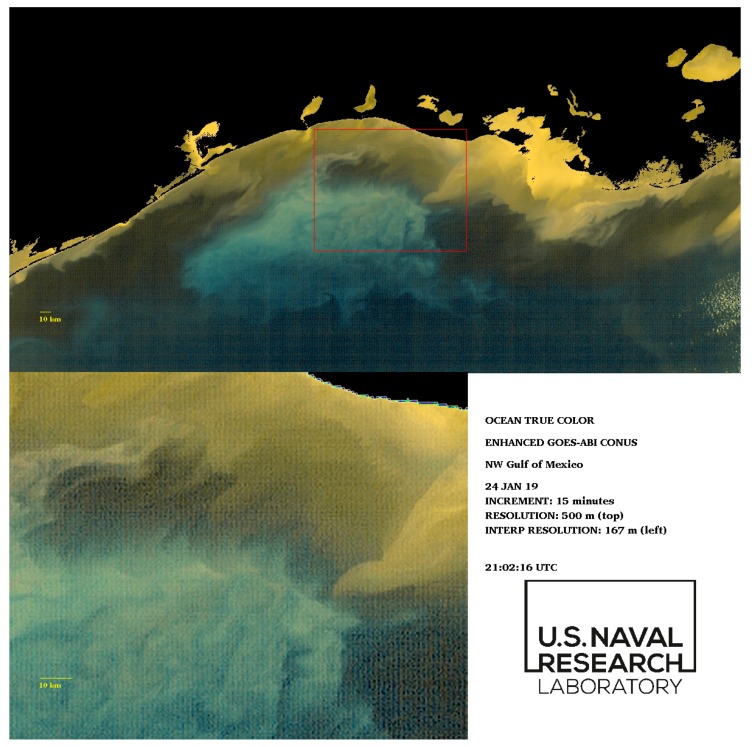
Final frame of enhanced GOES-ABI sequence for 24 January 2019, northern Gulf of Mexico. The red box (**top**) indicates frontal boundary zones; (**bottom**) the bottom panel is an interpolated increase in image resolution by a factor of 3. The full image sequence is available in Supplementary Materials Video S4.

The detailed movement of this color (and density) gradient is revealed using the enhanced-color GOES-ABI data (Figure 12 and Figure 13, also Supplementary Materials Video S4). At a reduced temporal frequency of 15 min (every 5 min are available), the data indicate substantial movement of the frontal boundary across the shelf over a 6-h interval. The low-salinity turbidity plume (inset Figure 12) appears to propagate towards the west at approximately ~0.5 m·s^−1^.

The combination of strong northerly winds and the cross-shelf buoyancy gradient are providing the circulation forcing that results in the accelerating surface currents suggested in the GOES-ABI enhanced-color image sequence. Further work will utilize numerical ocean circulation models to examine the physical setting of this particular image sequence. However, it is probable that there is some subduction of the denser, marine waters beneath the Atchafalaya turbidity plume as it propagates to the west. Such rapid vertical movements of water along frontal boundaries are a key characteristic of submesoscale processes. 

Characteristic features of CAO events in the northern Gulf of Mexico are the substantial drop in surface air temperature (by 10 °C or more) combined with northerly winds accelerating to ~10–15 m·s^−1^ [54]. This atmospheric forcing results in a significant thermal energy loss from the coastal ocean to the overlying atmosphere. There are two consequences of this air-sea interaction: (1) shallow areas of shelf will experience convective overturning of the water column, and this may result in ventilation of near-bottom turbidity layers and resuspended bottom sediments; and (2) shallower areas of shelf will experience a more rapid temperature decline than deeper areas (out to approximately 100 m depth) as a simple consequence of the total water volume per unit sea surface area subject to convective heat loss, and this will set up a cross-shelf sea surface temperature gradient (increasing SST with increasing bathymetry) [55]. This temperature difference will also be evident if a strong halocline, as would occur in a coastal river plume, prevents vertical overturn of the entire water column. The key dynamical question is whether or not the surface salinity gradient compensates for the cross-shelf temperature gradient, as a reversal of the surface cross-shelf density gradient may elicit an ageostrophic circulation response [39]. It appears likely that in this sequence (inset Figure 12 and Figure 13, Supplementary Materials Video S4) the temperature gradient is compensated by the salinity gradient and the marine waters (lighter colored turbidity) are subducting beneath the lower-salinity waters (darker colored turbidity). The enhanced-color GOES-ABI sequence provides a unique opportunity to examine these dynamics and build upon earlier work examining the coastal ocean’s optical and physical responses to synoptic atmospheric forcing [56]. 

## 4. Discussion

The traditional focus of “ocean color” remote sensing has been the development of inversion algorithms that estimate the surface concentration of specific constituents, often chlorophyll-*a* or suspended sediment concentration (in coastal areas), based on visible band reflectance data, and with each respective band confined to a narrow portion of the visible electromagnetic spectrum. The emphasis upon the space-borne detection of measurable sea surface quantities that have biogeochemical significance is certainly justified [57], however, the emerging constellation of polar-orbiting ocean color sensors as well as the data from GOCI are revealing the very high temporal frequency dynamics attendant to surface ocean optical gradients, particularly in coastal areas. Whereas the traditional ocean color sensors may resolve detailed constituent information about a particular coastal scene, the additional application of a non-traditional ocean sensor can extend this information into higher spatial or temporal frequency domains. This technique may then lead to the extraction of previously unavailable kinematic information, such as spatiotemporal expansion/propagation of frontal water mass boundaries or the synoptic pattern surface currents [58]. 

In this paper, we have demonstrated and described a method to convolve traditional ocean color remote sensing reflectance data from polar-orbiting sensors with spectrally-limited but temporally dense geostationary sensor data in order to observe these high frequency coastal processes. True color, or more properly, colorimetric analysis provides a useful frame of reference to facilitate this sensor merging technique. More generally, colorimetric analysis of water-leaving radiance data may facilitate improved utilization of digital color imaging from mobile devices [59], unmanned aerial vehicles [60], 3D printable devices [61], and emerging microsatellite platforms [62]. Furthermore, proper colorimetric analysis requires a hyperspectral radiant power distribution [32]; true color may thus be a useful frame of refence for emerging and planned hyperspectral satellite missions [63]. Hence, the very old idea that the apparent true color of natural waters is a useful and intrinsic oceanographic property [64] is worthy of reexamination [65]. 

## Supplementary Materials

The following are available online at https://zenodo.org/record/3364127#.XUy3_C2ZP2U, Video S1: 13 October 2018 GOES enhanced-color sequence; Video S2: expanded view–13 October 2018 GOES enhanced-color sequence; Video S3: 10 January 2019 GOES enhanced-color sequence; Video S3: 24 January 2019 GOES enhanced-color sequence.
